# Sorafenib Inhibits Tumor Growth and Improves Survival in a Transgenic Mouse Model of Pancreatic Islet Cell Tumors

**DOI:** 10.1100/2012/529151

**Published:** 2012-12-27

**Authors:** Volker Fendrich, Katja Maschuw, Johannes Rehm, Malte Buchholz, Julia P. Holler, Emily P. Slater, Detlef K. Bartsch, Jens Waldmann

**Affiliations:** ^1^Department of Surgery, University Hospital Giessen and Marburg, Baldinger Strasse, 35043 Marburg, Germany; ^2^Department of Gastroenterology and Endocrinology, University Hospital Giessen and Marburg, Marburg, Germany; ^3^Department of Surgery, University Hospital Giessen and Marburg, Giessen, Germany

## Abstract

*Background*. The purpose of the study was to evaluate Sorafenib (BAY 43-9006) derived receptor tyrosine kinase inhibition on tumor progression in murine islet cell tumors. Sorafenib is considered to be a potent inhibitor of tumor angiogenesis and neovascularization in various solid tumors. Rip1Tag2 mice were treated in two different groups according to the model of tumor progression: the early treatment group received vehicle or Sorafenib from 10 to 14 weeks of age and the late treatment group from week 12 until death. Tumor surface, tumor cell proliferation, and apoptosis were measured in both treatment groups to assess the in vivo effects of Sorafenib. Survival was recorded for the late treatment group. In the early treatment group Sorafenib led to a dramatic decrease in tumor volume compared to the control group. Apoptosis was significantly augmented and cell proliferation was inhibited. As a single therapy Sorafenib significantly improved survival in the late treatment group. *Conclusion*. Sorafenib may provide a new paradigm for the therapy of islet cell tumors.

## 1. Introduction

With an annual incidence of 1 per 100,000 individuals pancreatic neuroendocrine neoplasms (PNENs) occur rarely [1]. PNENs present as either functional (FPNENs) or nonfunctional pancreatic neuroendocrine neoplasms (NF-PNENs). FPNENs are associated with specific hormonal syndromes such as Zollinger-Ellisonsyndrome (ZES), organic hyperinsulinism, or very rarely with conditions related to a distinct hormone excess. The natural course of PNENs is highly variable. In general, complete tumor resection improves survival in patients with PNENs. Furthermore, in patients with advanced disease an aggressive surgical approach in combination with several treatment modalities for metastatic disease often results in long-term survival [2]. Survival is mainly determined by liver metastases that can be targeted by several approaches including surgery, chemoembolization, radiofrequency ablation, radiopeptide therapy (RPT), or selective internal radiation therapy (SIRT) [3]. Frequently, biotherapy with interferon or somatostatin analogues (SS) also is recommended for the treatment of metastatic disease achieving a tumor growth stabilization rather than inducing regression. Similar to SS analogues, this tumoristatic effect may result in improved survival, but at present has not been proven for PNENs [4, 5]. Therefore, new therapeutic strategies are urgently required. Recently, the mTor-inhibitor everolimus and the multitargeted sunitinib have been shown to increase overall survival in advanced neuroendocrine tumor patients in randomized controlled trials [6, 7]. Limitations of xenograft models to predict the effect of therapeutics may be resolved by introducing novel therapeutic approaches to an autochthonous model that recapitulates the multistep progression of the human disease. The Rip1Tag2 mouse model of pancreatic islet cell carcinoma offers such a model of spontaneous multistep tumorigenesis. Transgenic mice express the oncogene SV40 T antigen (Tag) under the control of the rat insulin gene promoter (Rip) and display different stages of tumor progression: onset of hyperproliferation, induction of angiogenesis and formation of solid tumors [8]. Rip1Tag2 mice succumb to insulinomas by the age of 14 weeks due to hypoglycemia. Tumor development in these mice proceeds through a series of discrete stages. Approximately 50% of the 400 pancreatic islets first start to proliferate, then a subset (25%) of pancreatic islets subsequently acquires the ability to enhance angiogenesis [9]. Again, a subset of these angiogenic islets (15%–20%) develops into benign, encapsulated lesions, and invasive carcinomas [10]. This multistage process suggests the sequential involvement of multiple genetic and epigenetic events in the progression from normal cells to tumors [9, 11, 12]. Therefore, this model provides an excellent approach to study new drug therapies in a preclinical setting [13, 14]. Since neovascularization represents a key element in the progression of islet cell tumors, inhibition of tumor-induced angiogenesis offers a rational therapeutic approach. VEGF-A (vascular endothelium-derived growth factor) with its alternative splicing forms -B, -C, -D, -E, and platelet-derived growth factor (PDGF) are essential promoters of adult angiogenesis [15]. They are expressed in both physiological and pathological settings [16, 17] and in tumor vascularization [18]. VEGF-A is upregulated in Rip1Tag2 tumorigenesis. The secreted form is sequestered in the extracellular matrix (ECM) and concomitantly released with the onset of angiogenesis by matrix metalloproteinases (MMPs) [19].

In the present study we evaluate the role of the small-molecule inhibitor of VEGFR-2 and PDGFR-*β* tyrosine kinase domains, Sorafenib, in Rip1Tag2 islet cell tumorigenesis with respect to the model-inherent biological steps of tumorigenesis: proliferation, angiogenesis, and vascularization. 

## 2. Materials and Methods

### 2.1. Mice

The generation of Rip1Tag2 mice as a model of pancreatic islet cell carcinogenesis has been reported previously [8]. The mice in the present study were males and females of the Rip1Tag2 transgenic mouse lineage bred in a C57BL/6J background. All experiments were approved by the local committees for animal care and use. Animals were maintained in a climate-controlled environment at 22°C, exposed to a 12 : 12 h light : dark cycle, fed standard laboratory chow, and given water ad libitum.

### 2.2. Genotyping

For genotyping genomic DNA was extracted from tail cuttings using the REDExtract-N-Amp Tissue PCR kit (Sigma-Aldrich, Saint Louis, MO, USA). The presence of Tag2 was verified by PCR. Primer sequences were TAG1—5′-GGA CAA ACC ACA ACT AGA ATG CAG—3′ and TAG2—5′-CAG AGC AGA ATT GTG GAG TGG—3′.

### 2.3. Drug Treatment

**Figure 1:**
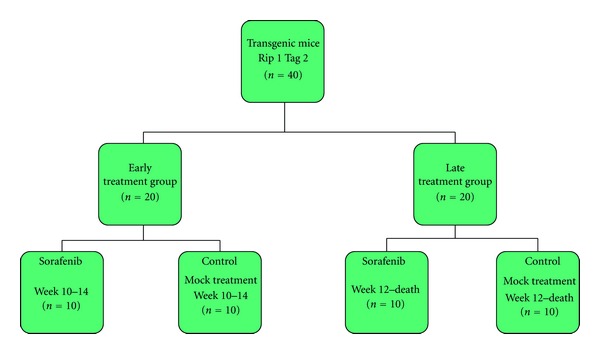
Study design for the assessment of Sorafenib in early and late treatment groups to analyze stage-dependent therapeutic effects.

The different study groups are explained in Figure 1. In the early treatment (ET) group mice were treated from 10 to 14 weeks of age and in the late treatment (LT) group from week 12 until death. Rip1Tag2 transgenic mice were randomly assigned to receive either (A) mock treatment or (B) single Sorafenib in the early and late treatment groups. When littermates were available for drug treatment, only the first mouse was randomly assigned to one of the 2 given treatment groups; the second littermate was then assigned to the “matched” control arm, and so forth. This scheme was applied to obtain the highest degree of consistency and to avoid randomization bias as far as possible.

Sorafenib was dissolved in 5% Cremophor EL (Sigma-Aldrich) and 5% ethanol 99.8% at 4-fold of the highest dose, foil wrapped and stored at room temperature. The 4-fold stock solution was prepared every 3 days. Final dosing solutions were prepared immediately prior to application by diluting the stock solution with distilled water to obtain final concentrations of 750 *μ*g/200 *μ*L per mouse and treatment (30 mg/kg per day) was administered by intraperitoneal (i.p.) injection. This dose was chosen according to studies from Wilhelm et al. [20]. 

### 2.4. Necropsy and Assessment of Islet Cell Tumor Growth

After the drug treatment, mice in the ET group were euthanized and the pancreas was removed, weighed, and screened for grossly visible tumors and either preserved in 10% formalin solution (Sigma-Aldrich) for histology or processed for RNA extraction (see below). Sections were evaluated as described previously [21]. Tumor volume was measured as previously described [22].

### 2.5. Immunostaining

For immunolabeling formalin-fixed and paraffin-embedded archived tumor samples and corresponding normal tissue were stained as previously described [21]. Primary antibodies were *α*-Caspase 3 (host rabbit, 1 : 200, Cell Signaling, Danvers, MA, USA) and *α*-BrdU (host mouse, Zymed, San Francisco, CA, USA). Briefly, slides were heated to 60°C for 1 h, deparaffinized using xylene, and hydrated by a graded series of ethanol washes. Antigen retrieval was accomplished by microwave heating in 10 mM sodium citrate buffer, pH 6.0, for 10 min. For immunohistochemistry endogenous peroxidase activity was quenched by 10 min incubation in 3% H_2_O_2_. Nonspecific binding was blocked with 10% serum. Sections were then incubated with primary antibodies overnight at 4°C. For immunohistochemistry, bound antibodies were detected using the avidin-biotin-complex (ABC) peroxidase method (ABC Elite Kit, Vector Labs, Burlingame, CA, USA). Final staining was developed with the Sigma FAST DAB peroxidase substrate kit (Sigma, Deisenhofen, Germany). For BrdU staining, tissues were prepared as above, except that mice were injected intraperitoneally with 100 mg of bromodeoxyuridine (BrdU) per gram of body weight 2 hours prior to sacrifice. The immunohistochemical results were scored as follows: negative = less than 5% cells positive; + = <30% cells positive; ++ = >30% cells positive. BrdU and caspase-3 positive cells were counted by manual assessment within defined 10x fields of view (*n* = 3/section; 3 sections analyzed/animal). BrdU and caspase-3-positive cells were counted as a fraction of total nuclei within a region.

### 2.6. RNA Extraction and Real-Time RT-PCR

Normal (from nontransgenic C57BL/6J) and hyperplastic islets (from 5-week-old Rip1Tag2 mice) were collected as previously described [13]. Angiogenic islets were isolated from 8-week-old Rip1Tag2 mice by collagenase digestion and selected based on their red, hemorrhagic appearance [13]. Tumors were microdissected from the excised pancreas of 10- and 14-week-old Rip1Tag2 mice and the exocrine tissue was carefully removed. RNA extraction and cDNA synthesis were performed as previously described [21]. All PCRs were carried out on a 7500 Real-Time PCR System (Applied Biosystems, Foster City, CA, USA) over 40 cycles, with denaturation for 15 sec at 95°C and combined annealing/extension at 60°C for 1 min. Following an activation step at 95°C for 10 min, determination of mRNA expression was performed over 40 cycles with 15 seconds of denaturation at 95°C and annealing/extension/data acquisition at 60°C for 60 seconds using the Power SYBR Green PCR kit (Applied Biosystems). Primer sequences are available upon request. Relative-fold mRNA expression levels were determined using the 2(−ΔΔCt) method [23]. All reactions were performed in triplicate and results are presented as means with standard errors. 

### 2.7. Statistical Analysis

Survival curves were calculated using the Kaplan-Meier method. Log-rank test was applied to identify significant differences. Noncategorical parameters were were analyzed by a Mann-Whitney *U* test. Comparisons of more than two groups were made by a one-way ANOVA with post hoc Holm-Sidak analysis for pairwise comparisons and comparisons versus control and by Kruskal-Wallis one-way analysis of variance. *P* values < 0.05 were considered statistically significant. Data were analyzed using SPSS software (Version 14; SPSS, Inc., Chicago, IL, USA).

## 3. Results

**Figure 2:**
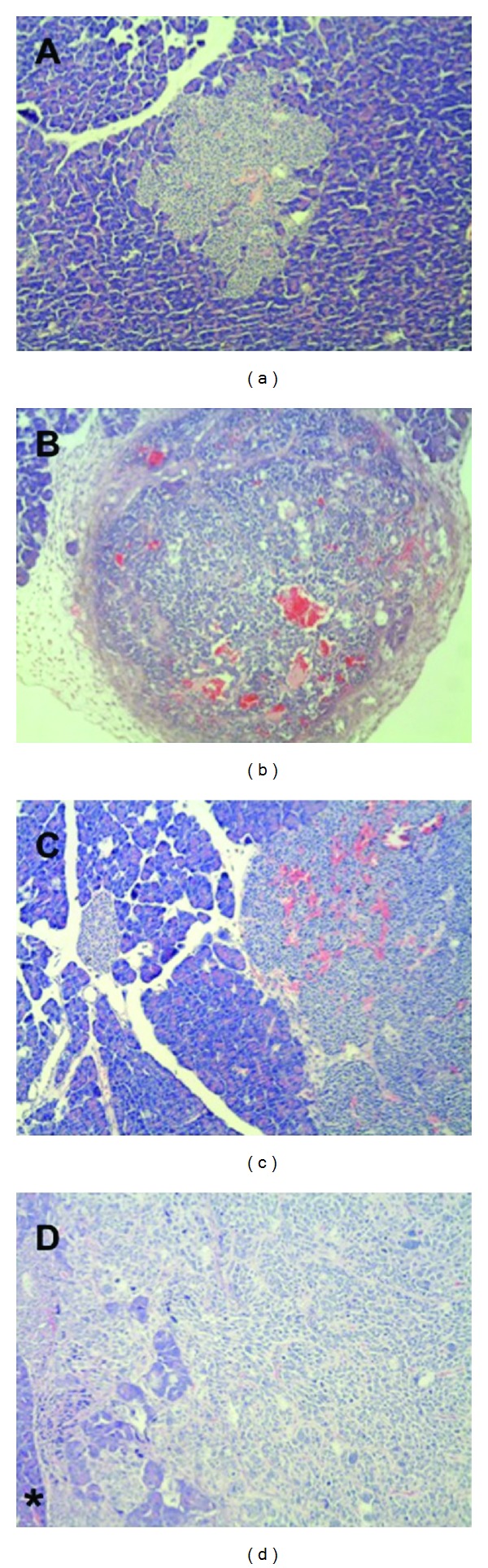
Rip1Tag2 mice developed (a) islet cell hyperplasia, (b) angiogenetic islets marked by the increase of red blood cells throughout the islet, and (c) islet cell tumors, invading surrounding structures (asterisk in (d)).

The treatment was well tolerated by the Rip1Tag2 mice. No deaths nor obvious side effects were observed. In the Rip1Tag2 mouse cohort of both the ET and the LT groups (see also Figure 1) the development of islet cell hyperplasia (Figure 2(a)), angiogenetic islets (Figure 2(b)) and islet cell tumors (Figure 2(c)) developed as previously reported when compared to wild type control mice (Figure 2(d)) [5].

### 3.1. Effect of Sorafenib on Tumor Growth and Apoptosis in the Intervention Group

**Figure 3:**
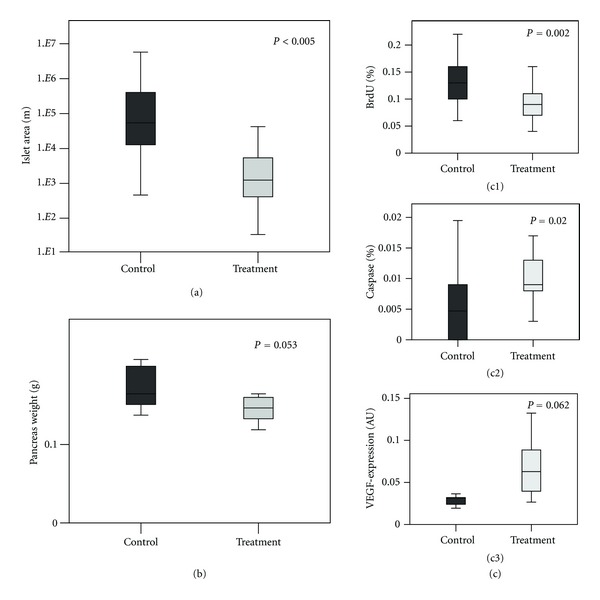
Effects of Sorafenib in the early treatment group on tumor size (measured by the tumor area in *μ*m^2^) and on pancreas weight (mg) of Rip1Tag2 mice assessed by manual measurement. (a) Boxplot for the islet cell area; (b) Boxplot for the pancreas weight (mg). (c) Treatment with Sorafenib significantly augmented apoptosis and inhibited proliferation. (c (c1 and c2)) Boxplots showing percentage (%) of caspase-3 positive and BrdU positive cells in mice from the early treatment group induced by Sorafenib. (c (c3)) Treatment with Sorafenib significantly increased VEGF-expression. VEGF-expression in Sorafenib treated and control mice assessed by RT-PCR.

Treatment with Sorafenib led to a dramatic, highly statistically significant reduction in tumor size (436189 *μ*m^2^ versus 6095 *μ*m^2^, *P* = 0.002), corresponding to a tumor size reduction of 98.6%. In line with the tumor size reduction, the pancreas weight of Sorafenib-treated mice decreased (0.145 ± 0.19 g versus 0.192 ± 0.15 g, *P* = 0.053), but failed to reach statistical significance (see also Figure 3(a)). To address the effect on tumor biology, apoptosis and tumor cell proliferation were both evaluated in Sorafenib-treated mice compared to control mice. Apoptosis, as measured by activated caspase-3 immunostaining, was significantly enhanced in response to Sorafenib compared to mock-treated mice (0.011 ± 0.007% versus 0.007 ± 0.006%; *P* = 0.020) (see also Figure 3(c2)). In addition to enhancing apoptosis Sorafenib caused a corresponding significant decrease in tumor cell proliferation in the ET group (0.094 ± 0.430% versus 0.137 ± 0.400%; *P* < 0.005) as measured by BrdU-incorporation (see also Figure 3(c1)). In response to Sorafenib VEGF-expression increased, although the difference was not quite statistically significant (see Figure 3(c3)).

### 3.2. Effect of Sorafenib on Survival of Rip1Tag2 Mice

**Figure 4:**
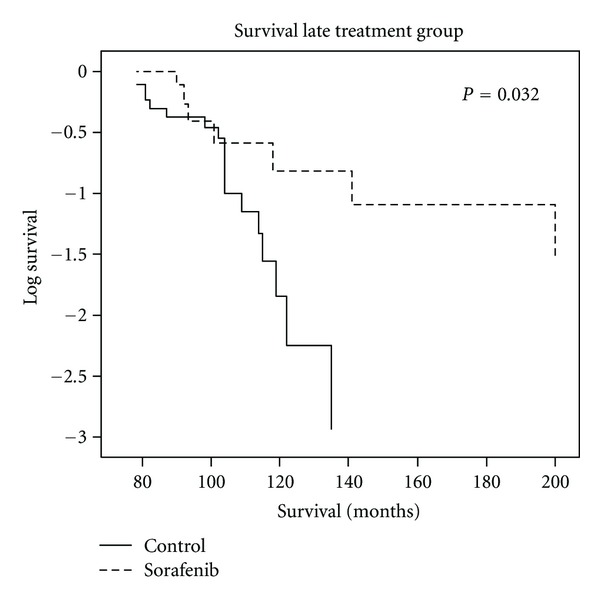
Sorafenib prolongs survival in Rip1Tag2 mice. Kaplan-Meier curve and log rank-analysis of Rip1Tag2 mice treated with Sorafenib or mock treatment. Note the significant increased survival in mice treated with Sorafenib (137.7 ± 17.8 d versus 102.8 ± 4.2 d; *P* = 0.032 log rank test).

Mice in the LT group (see Figure 1) that received Sorafenib showed a significantly prolonged survival (137.7 ± 17.8 d versus 102.8 ± 4.2 d; *P* = 0.032 log rank test), corresponding to a survival benefit of almost 30 days (see also Figure 4).

## 4. Discussion

Rip1Tag2 transgenic mice provide a model for the molecular mechanisms of multistage tumorigenesis in islet cell tumors [24]. Bergers and colleagues [25, 26] have used the Rip1Tag2 model to assess the effectiveness of angiogenesis and matrix metalloproteinases inhibitors. The response to medical treatment seemed to be highly dependent on the stage of disease. In an expression screen of ~thirty growth factors and their receptors, insulin-like growth factor-2 (IGF-2) has been shown to be upregulated in tumors when compared to normal islets in Rip1Tag2 mice [27–30]. Although the expression of large T-antigen, as seen in *β*-cells of Tag transgenic mice, is not known to occur in human islet tumors, Wulbrand and colleagues demonstrated that members of the IGF family and their corresponding receptors and binding proteins are important in carcinogenesis of several tumors, including human pancreatic endocrine tumors [30, 31]. 

At the intermediate stage of tumor progression we have demonstrated that Sorafenib induces growth inhibition by an induction of apoptosis and reduction of proliferation. Suppressed tumor growth was associated with an upregulation of VEGF that is known to be a systemic effect of Sorafenib promoted by the VEGF-receptor blockage. This is also observed in renal cell carcinoma patients treated with Sorafenib and a greater than 2-fold increase seemed to predict a superior response [32]. In Rip1Tag2 mice, specific inhibition of VEGF, MMPs, soluble VEGFR, or VEGFR signal transduction is known to repress tumor angiogenesis [33, 34]. Another key element for tumor proliferation and angiogenesis is the activation of the Raf/Mek/ERK pathway. Tumor cells harboring gain-of-function mutations in B-Raf reactivate the extracellular kinase pathway for proliferation and tumor angiogenesis. The development of Rip1Tag2 tumors is promoted by B-Raf-dependent stromal response and tumor progression. Dual molecular agents are promising tools to silence tumor growth on the basis of angiogenesis and proliferation. The small molecular inhibitor of VEGFR 2-, 3-kinases and the c KIT receptor of the stem cell factor, Sorafenib (BAY 43-9006), exhibits both of these characteristics. Simultaneously targeting VEGFR-derived and Raf/Mek/Erk pathways, Sorafenib is an attractive multifunctional agent for the treatment of solid tumors. It has already been tested in advanced renal cell carcinoma and hepatocellular carcinomas [35].

During the angiogenic switch and formation of islet tumors, administration of Sorafenib significantly increased apoptosis while decreasing proliferation and, correspondingly, tumor size. The predominant effect of Sorafenib is probably rooted in the multidimensional character of the drug itself, addressing pathways of tumorigenesis that are active and relevant for Rip1Tag2 tumor progression such as VEGFR 2,3 kinases and Raf/Mek/ERK [33, 34]. The present study is supported by results from the Hanahan group who recently reported Brivanib, a dual FGF/VEGF-inhibitor for the first- and second-line therapy in PNETs [36].

In summary, for the first time the tumor suppressive effects of the multifunctional inhibitor of tyrosine kinases, Sorafenib, could be demonstrated in islet cell tumors of Rip1Tag2 mice through increased apoptosis, decreased proliferation, and prolonged survival. This supports a potential application for advanced pancreatic neuroendocrine tumors and may extend the indication for Sorafenib that has been proven to prolong survival in renal cell and hepatocellular carcinoma.

The observations of the present study have obvious clinical implications and seem to justify clinical trials for Sorafenib-induced tumor suppressive therapy for human pancreatic neuroendocrine neoplasms (PNENs).

## References

[1] Öberg K, Eriksson B (2005). Endocrine tumors of the pancreas. *Best Practice & Research Clinical Gastroenterology*.

[2] Fendrich V, Langer P, Celik I (2006). An aggressive surgical approach leads to long-term survival in patients with pancreatic endocrine tumors. *Annals of Surgery*.

[3] Metz DC, Jensen RT (2008). Gastrointestinal neuroendocrine tumors: pancreatic endocrine tumors. *Gastroenterology*.

[4] Plockinger U, Wiedenmann B (2007). Biotherapy. *Best Practice & Research: Clinical Endocrinology & Metabolism*.

[5] Rinke A, Müller HH, Schade-Brittinger C (2009). Placebo-controlled, double-blind, prospective, randomized study on the effect of octreotide LAR in the control of tumor growth in patients with metastatic neuroendocrine midgut tumors: a report from the PROMID Study Group. *Journal of Clinical Oncology*.

[6] Raymond E, Dahan L, Raoul JL (2011). Sunitinib Malate for the treatment of pancreatic neuroendocrine tumors. *New England Journal of Medicine*.

[7] Yao JC, Shah MH, Ito T (2011). Everolimus for advanced pancreatic neuroendocrine tumors. *New England Journal of Medicine*.

[8] Hanahan D (1985). Heritable formation of pancreatic *β*-cell tumours in transgenic mice expressing recombinant insulin/simian virus 40 oncogenes. *Nature*.

[9] Folkman J, Watson K, Ingber D, Hanahan D (1989). Induction of angiogenesis during the transition from hyperplasia to neoplasia. *Nature*.

[10] Lopez T, Hanahan D (2002). Elevated levels of IGF-1 receptor convey invasive and metastatic capability in a mouse model of pancreatic islet tumorigenesis. *Cancer Cell*.

[11] Peri AK, Wilgenbus P, Dahl U, Semb H, Christofori G (1998). A causal role for E-cadherin in the transition from adenoma to carcinoma. *Nature*.

[12] Perl AK, Dahl U, Wilgenbus P, Cremer H, Semb H, Christofori G (1999). Reduced expression of neural cell adhesion molecule induces metastatic dissemination of pancreatic *β* tumor cells. *Nature Medicine*.

[13] Parangi S, O’Reilly M, Christofori G (1996). Antiangiogenic therapy of transgenic mice impairs de novo tumor growth. *Proceedings of the National Academy of Sciences of the United States of America*.

[14] Inoue M, Hager JH, Ferrara N, Gerber HP, Hanahan D (2002). VEGF-A has a critical, nonredundant role in angiogenic switching and pancreatic *β* cell carcinogenesis. *Cancer Cell*.

[15] Ferrara N (1999). Vascular endothelial growth factor: molecular and biological aspects. *Current Topics in Microbiology and Immunology*.

[16] Ferrara N, Chen H, Davis-Smyth T (1998). Vascular endothelial growth factor is essential for corpus luteum angiogenesis. *Nature Medicine*.

[17] Carmeliet P (2000). VEGF gene therapy: stimulating angiogenesis or angioma-genesis?. *Nature Medicine*.

[18] Kerbel RS (2000). Tumor angiogenesis: past, present and the near future. *Carcinogenesis*.

[19] Christofori G, Naik P, Hanahan D (1995). Vascular endothelial growth factor and its receptors, flt-1 and flk-1, are expressed in normal pancreatic islets and throughout islet cell tumorigenesis. *Molecular Endocrinology*.

[20] Wilhelm SM, Adnane L, Newell P, Villanueva A, Llovet JM, Lynch M (2008). Preclinical overview of sorafenib, a multikinase inhibitor that targets both Raf and VEGF and PDGF receptor tyrosine kinase signaling. *Molecular Cancer Therapeutics*.

[21] Fendrich V, Waldmann J, Feldmann G (2009). Unique expression pattern of the EMT markers Snail, Twist and E-cadherin in benign and malignant parathyroid neoplasia. *European Journal of Endocrinology*.

[22] Fendrich V, Wiese D, Waldmann J (2011). Hedgehog inhibiton with the orally bioavailable Smo antagonist LDE 225 represses tumor growth and prolongs survival in a transgenic mouse model of islet cell neoplasms. *Annals of Surgery*.

[23] Livak KJ, Schmittgen TD (2001). Analysis of relative gene expression data using real-time quantitative PCR and the 2-ΔΔCT method. *Methods*.

[24] Bergers G, Javaherian K, Lo KM, Folkman J, Hanahan D (1999). Effects of angiogenesis inhibitors on multistage carcinogenesis in mice. *Science*.

[25] Bergers G, Brekken R, McMahon G (2000). Matrix metalloproteinase-9 triggers the angiogenic switch during carcinogenesis. *Nature Cell Biology*.

[26] Christofori G, Naik P, Hanahan D (1994). A second signal supplied by insulin-like growth factor II in oncogene- induced tumorigenesis. *Nature*.

[27] Porcu P, Ferber A, Pietrzkowski Z (1992). The growth-stimulatory effect of simian virus 40 T antigen requires the interaction of insulinlike growth factor 1 with its receptor. *Molecular and Cellular Biology*.

[28] Petrik J, Pell JM, Arany E (1999). Overexpression of insulin-like growth factor-II in transgenic mice is associated with pancreatic islet cell hyperplasia. *Endocrinology*.

[29] Wulbrand U, Wied M, Zöfel P, Göke B, Arnold R, Fehmann HC (1998). Growth factor receptor expression in human gastroenteropancreatic neuroendocrine tumours. *European Journal of Clinical Investigation*.

[30] Wulbrand U, Remmert G, Zöfel P, Wied M, Arnold R, Fehmann HC (2000). mRNA expression patterns of insulin-like growth factor system components in human neuroendocrine tumours. *European Journal of Clinical Investigation*.

[31] Bergers G, Brekken R, McMahon G (2000). Matrix metalloproteinase-9 triggers the angiogenic switch during carcinogenesis. *Nature Cell Biology*.

[32] Flaherty KT (2007). Sorafenib in renal cell carcinoma. *Clinical Cancer Research*.

[33] Compagni A, Wilgenbus P, Impagnatiello MA, Cotten M, Christofori G (2000). Fibroblast growth factors are required for efficient tumor angiogenesis. *Cancer Research*.

[34] Sobczak I, Galabova-Kovacs G, Sadzak I, Kren A, Christofori G, Baccarini M (2008). B-Raf is required for ERK activation and tumor progression in a mouse model of pancreatic *β*-cell carcinogenesis. *Oncogene*.

[35] Fujita M, Hayashi I, Yamashina S, Fukamizu A, Itoman M, Majima M (2005). Angiotensin type 1a receptor signaling-dependent induction of vascular endothelial growth factor in stroma is relevant to tumor-associated angiogenesis and tumor growth. *Carcinogenesis*.

[36] Inoue M, Hager JH, Ferrara N, Gerber HP, Hanahan D (2002). VEGF-A has a critical, nonredundant role in angiogenic switching and pancreatic *β* cell carcinogenesis. *Cancer Cell*.

